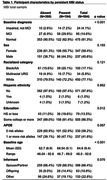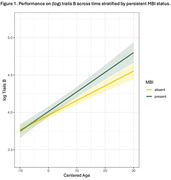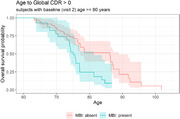# Mild Behavioral Impairment is associated with incident cognitive decline among dementia‐free, racially diverse older adults: Data from the African Americans Fighting Alzheimer’s in Midlife (AA‐FAIM) study

**DOI:** 10.1002/alz.089933

**Published:** 2025-01-03

**Authors:** Barbara L. Fischer, Carol A. Van Hulle, Derek L. Norton, Mary F. Wyman, Gilda E. Ennis, Megan L. Zuelsdorff, Nikolaus H Lambrou, Shenikqua Bouges, Diane Carol Gooding, Carey E. Gleason

**Affiliations:** ^1^ Department of Neurology, University of Wisconsin‐Madison, School of Medicine and Public Health, Madison, WI USA; ^2^ Geriatric Research, Education and Clinical Center (GRECC), William S. Middleton Memorial Veterans Hospital, Madison, WI USA; ^3^ Wisconsin Alzheimer’s Institute, University of Wisconsin‐Madison, School of Medicine and Public Health, Madison, WI USA; ^4^ Wisconsin Alzheimer’s Disease Research Center, University of Wisconsin‐Madison, SMPH, Madison, WI USA; ^5^ Wisconsin Alzheimer’s Disease Research Center, University of Wisconsin School of Medicine and Public Health, Madison, WI USA; ^6^ Wisconsin Alzheimer’s Institute, University of Wisconsin‐Madison, School of Medicine and Public Health, Madison, WI USA; ^7^ Department of Biostatistics and Medical Informatics, University of Wisconsin‐Madison, Madison, WI USA; ^8^ University of Wisconsin School of Medicine and Public Health, Department of Psychiatry, Madison, WI USA; ^9^ Wisconsin Alzheimer’s Disease Research Center, University of Wisconsin, School of Medicine and Public Health, Madison, WI USA; ^10^ Madison VA GRECC, William S. Middleton Memorial Hospital, Madison, WI USA; ^11^ Department of Medicine, University of Wisconsin‐Madison School of Medicine and Public Health, Madison, WI USA; ^12^ School of Nursing, University of Wisconsin‐ Madison, Madison, WI USA; ^13^ Wisconsin Alzheimer’s Institute, University of Wisconsin School of Medicine and Public Health, Madison, WI USA; ^14^ UW‐Madison, Madison, WI USA; ^15^ Division of Geriatrics and Gerontology, University of Wisconsin‐Madison, School of Medicine and Public Health, Madison, WI USA; ^16^ Departments of Psychology and Psychiatry, University of Wisconsin‐Madison School of Medicine & Public Health, Madison, WI USA; ^17^ Department of Psychology, University of Wisconsin‐Madison, College of Letters & Science, Madison, WI USA; ^18^ William S. Middleton Memorial Veterans Hospital, Madison, WI USA; ^19^ Wisconsin Alzheimer’s Institute, University of Wisconsin‐Madison School of Medicine and Public Health, Madison, WI USA; ^20^ Alzheimer’s Disease Research Center, University of Wisconsin‐Madison School of Medicine and Public Health, Madison, WI USA

## Abstract

**Background:**

Mild behavioral impairment (MBI) is associated with all‐cause dementia. Little is known about MBI’s effects on cognitive function among individuals in earlier disease stages, particularly in racially diverse samples. We examined relationships between MBI and cognitive decline in a richly characterized sample of white and African American (AA) older‐middle aged adults. AA participants were enrolled in African Americans Fighting Alzheimer’s in Midlife (AA‐FAIM), an ancillary study of the Wisconsin Disease Research Center’s (WADRC) Clinical Core.

**Method:**

Analytic sample included participants without dementia, with ≥1 study visits and measures of cognitive/clinical function. A baseline MBI rating (present/absent) was derived using study partners’ report of neuropsychiatric symptoms at two consecutive visits, (i.e., two consecutive positive MBI scores = MBI present). Cognitive outcomes included measures of speed, mental flexibility and memory.

**Result:**

Table 1 provides characteristics by MBI status for white (N = 450), AA (N = 100), and other underrepresented group (URG) (N = 34) participants. In the full sample, baseline MBI significantly moderated cognitive trajectory on Trails B, though not on any other tasks. Participants with MBI displayed worse trajectory of decline on Trails B relative to those without MBI (Figure 1). Additionally, MBI at baseline significantly predicted progression to cognitive impairment CDR >0 (HR est: 2.84; HR 95% CI: 1.68 — 4.81; p = 0.0001) (Figure 2). Stratified analysis in a smaller AA subgroup showed a similar pattern but was not significant.

**Conclusion:**

MBI associated with more adverse cognitive trajectory and predicted incident cognitive decline in older, middle aged participants without dementia. Our findings highlight the importance of identifying MBI as a possible target for intervention in early disease stages. A fifth of our sample (21.5%) identified as Black/URG. Still, ongoing work in inclusive cohorts is needed to better understand MBI in under‐represented groups.